# Investigating factors affecting the interval between a burn and the start of treatment using data mining methods and logistic regression

**DOI:** 10.1186/s12874-021-01270-5

**Published:** 2021-04-14

**Authors:** Touraj Ahmadi-Jouybari, Somayeh Najafi-Ghobadi, Reza Karami-Matin, Saeid Najafian-Ghobadi, Khadijeh Najafi-Ghobadi,

**Affiliations:** 1 Clinical Research Development Center, Imam Khomeini and Mohammad Kermanshahi Hospitals, Kermanshah University of Medical Sciences, Kermanshah, Iran; 2 Department of Industrial Engineering, Faculty of Engineering, Kermanshah Branch, Islamic Azad University, Kermanshah, Iran; 3 Burn Unit of Imam Khomeini Hospital Kermanshah University of Medical Sciences, Kermanshah, Iran; 4 Department of Industrial Engineering, Faculty of Engineering, West Tehran Branch, Islamic Azad University, Tehran, Iran

**Keywords:** Burns, Start of treatment, Random Forest, Decision tree, Support Vector Machine, Logistic regression

## Abstract

**Background:**

Burn is a tragic event for an individual, the family, and community. It can cause irreparable physical, mental, economic, and social injury. Researches well documented that a quick visit to a healthcare center can greatly reduce burn injuries. Therefore, the aim of this study is to identify the effective factors in the interval between a burn and start of treatment in burn patients by comparing three classification data mining methods and logistic regression.

**Methods:**

This cross-sectional study conducted on 389 hospitalized patients in Imam Khomeini Hospital of Kermanshah city since 2012 to 2015. The data collection instrument was a three-part questionnaire, including demographic information, geographical information, and burn information. Four classification methods (decision tree (DT), random forest (RF), support vector machine (SVM) and logistic regression (LR)) were used to identify the effective factors in the interval between burn and start of treatment (less than two hours and equal or more than two hours).

**Results:**

The mean total accuracy of all models is higher than 0.8. The DT model has the highest mean total accuracy (0.87), sensitivity (0.44), positive likelihood ratio (14.58), negative predictive value (0.89) and positive predictive value (0.71). However, the specificity of the SVM model and RF model (0.99) was higher than other models, and the mean negative likelihood ratio (0.98) of the SVM model are higher than other models.

**Conclusions:**

The results of this study shows that DT model performed better that data mining models in terms of total accuracy, sensitivity, positive likelihood ratio, negative predictive value and positive predictive value. Therefore, this method is a promising classifier for investigating the factors affecting the interval between a burn and the start of treatment in burn patients. Also, key factors based on DT model were location of transfer to hospital, place of occurrence, time of accident, religion, history and degree of burn, income, province of residence, burnt limbs and education.

## Background

The burn is a tragic event for individuals, families, and communities. It can cause irreparable physical, psychological, economic, and social injury. The burn is one of the most important diseases all over the world [1]. Burns after traffic accidents, falls and interpersonal violence are the fourth most common injuries [2]. World Health Organization has estimated that 265,000 deaths occur each year due to firing and burn with boiling water, electric burn and other injuries. More than 96 % of burn deaths occur in low- and middle-income countries [3]. Based on standardized age in 2017, the regions with the highest rate of burn were Eastern Europe with 303 per 100,000, Central Asia with 298 per 100,000 and South Latin America with 226 per 100,000 [4]. Burns are the third leading cause of death in the United States after accident and drowning, and the sixth leading cause of death in Iran [5]. It is a well-known fact that the most important factors in the mortality of burn patients are age, inhalation of burns and percentage of the total body surface area (TBSA) [6, 7].

Epidemiological studies conducted in emergency centers in Iran and other countries indicate that burns are one of the most important public health problems that lead to death, disability, pain, physical, mental and economic problems [8–10]. Evidence have reported that burn injuries cause significant limitations that go far beyond physical issues and affect people’s emotional, social, and family relationships [11–13]. Therefore, the shorter the interval between burns and the start of treatment and the sooner the patient goes to the medical center, the fewer these complications will be [14, 15]. Hence, the present study was conducted to identify the factors affecting the interval between burns and the start of treatment in patients referred to the Imam Khomeini Hospital of Kermanshah city during the years 2012–2015.

To achieve this purpose, we compared the performance of three well-known data mining models including RF, DT, and SVM with the LR as a classical technique. Recently, a growing number of studies, especially in the field of public and medical health, has compared the accuracy of traditional classifiers with data mining methods. Some studies revealed that data mining techniques have higher accuracy and lower error rates than the traditional models [16–18] and some others found better performance for traditional methods [19–21]. To the best of our knowledge, there is not any study that compared the traditional classifiers with the data mining classifiers like RF, SVM, and DT for predicting the factors affecting the interval between burns and the start of treatment.

## Methods

### Dataset

The present study was a cross-sectional descriptive-analytical study to investigate the factors affecting the interval between a burn and the start of treatment in burn patients in Imam Khomeini Hospital of Kermanshah city from 2012 to 2015. The data gathering instrument was a three-part questionnaire. We used information on 18 risk factors that appear to be effective in the interval between burn time and the start of treatment. These risk factors included: age, gender, marital status, occupational status, place of residence, education, religion, income, burn percentage, history of burns, time of the accident, place of occurrence, burnt limbs, province of residence, location of transfer to hospital, the cause of burn, type burn, degree of burn. All information was collected and recorded by a trained investigator from information in the patient file, interviews with patients and relatives. In this study, according to the burn specialist opinion, 120 min was considered as a cut-off point for the interval between a burn and the start of treatment and defined this variable as a binary variable (less than 120 min and equal or more than 120 min) [14, 22–24].

### Data pre‐processing and dealing with missing values

Before the model application, the missing data and outliers were checked consistently. The missing data across all variables for the dataset ranged from 0 to 15.4 %. The highest missing data were time of accident (15.4 %). Variables with missing values were imputed using CART regression trees and their mode [25]. We used Anomaly detection to indicate outliers. Anomaly detection provides very important and critical information for outlier detection in various applications [26]. By considering value 2 as a threshold for Anomaly detection, there were no outlier records [27]. For a better interpretation of the results, associate degrees, bachelor and master were combined with a single “college education” group for the analysis of variable education. The demographic statistics and summaries of the variables in the data analysis are shown separately for the two groups of response variables in Table 1.

**Table 1:** Summary of discrete variables

Variables	< 120	≥ 120
	N (%)	N (%)
**Gender**		
Men	180 (56.4)	31 (44.3)
Women	139 (43.6)	39 (55.7)
**Place of residence**		
Town	184 (57.7)	27 (38.6)
Village	135 (42.3)	43 (61.4)
**History of burns**		
Yes	56 (17.6)	2 (2.9)
No	263 (82.4)	68 (97.1)
**Religion**		
Shia	185 (58.0)	54 (77.1)
Sunni	99 (31.0)	12 (17.1)
Other	35 (10.9)	4 (5.7)
**Burn percentage**		
<25%	161 (50.5)	38 (54.3)
25-50%	104 (32.6)	23 (32.9)
>50%	54 (16.9)	9 (12.9)
**Age**		
<14	164 (51.4)	22(31.4)
14-35	83 (26.0)	29(41.4)
>35	72 (22.6)	19(27.1)
**Education**		
Illiterate	184 (57.7)	32 (45.7)
High school	83 (26.0)	24 (34.3)
Diploma	40 (12.5)	11 (15.7)
College education	12 (3.8)	3 (4.3)
**cause of burn**		
Fire or flame	257 (80.6)	49 (70.0)
Scald	50 (15.7)	19 (27.1)
Chemical	3 (0.9)	0
Electrical	6 (1.9)	2 (2.9)
Contact	3 (0.9)	0
**Burnt limbs**		
Upper limb	52 (16.3)	21 (30.0)
Lower limb	115 (36.1)	12 (17.1)
Combinatorial	152 (47.6)	37 (52.9)
**Occupational status**		
Unemployed	254 (79.6)	53 (75.7)
Employed	65 (20.4)	17 (24.3)
**Marital status**		
Single	151 (47.3)	33 (47.1)
Married	168 (52.7)	37 (52.9)
**Type burn**		
Intentional	57 (17.9)	12 (17.1)
Accidental	262 (82.1)	58 (82.9)
**Income (US$)**		
<200	177(55.5)	20 (28.6)
200-319	123(38.6)	45 (64.3)
≥320	19(6.0)	5 (7.1)
**Location of transfer to hospital**		
From downtown	104 (32.6)	9 (12.9)
Of the subsidiary cities	177 (55.5)	42 (60.0)
From other provinces	38 (11.9)	19 (27.1)
**Place of occurrence**		
Home	174 (54.5)	54 (77.1)
Work placement	117 (36.7)	9 (12.9)
Other	28 (8.8)	7 (10.0)
**Degree of burn**		
First-degree	82 (25.7)	0
Second-degree	9 (2.8)	0
Third-degree	126 (39.5)	22 (31.4)
Combinatorial	102 (32.0)	48( 68.6)
**Time of accident**		
Morning(6:00-11:59)	142 (44.5)	23 (32.9)
Noon(12.00-15:59)	50 (15.7)	13 (18.6)
Evening(16:00-18:59)	62 (19.4)	19 (27.1)
Night(19:00-23:59)	61 (19.1)	14 (20.0)
Midnight(24:00-5:59)	4 (1.3)	1 (1.4)
**Province of residence**		
Other provinces	78(24.5)	21(30.0)
Kermanshah	241(75.5)	49(70.0)

### Classification models

In this paper, DT, RF, SVM and logistics regression models were used to identify the factors involved in interval between burn and start of treatment in burn patients referred to Imam Khomeini Hospital from 2012 to 2015. Each of the used methods will be described briefly.

One of the simplest and most common classification techniques is the DT. The main goal of the DT (like other classification techniques) is to build a model that can predict variable response values. The DT is made up of nodes and partitions. The construction of the tree begins with the presence of all training data in the first node. Then, the first partition divides the data into two or more daughter nodes based on a predictor variable. The DT has three types of nodes:

Root node: It has no input branch and the number of its output branches can be zero or more.

Intermediate node: It consists of one input branch and two or more output branches.

Final node or leaf: It consists of one input branch and had no output branch.

In the DT, a category is allocated to each final node [28].

RF method is a non-parametric statistical method (free model) for classification analysis and regression analysis using recursive partitioning algorithm. The RF algorithm uses a set of classified trees [29]. This method is very effective in selecting a set of predictive variables, which best express the phenotypes of the disease. RF method is also useful when predictor variables are nonlinearly related to disease because they do not assume any constraints on the relationship between predictor and response variables. These methods are often compatible with genetic heterogeneities, so that individual models are automatically fitted to subsets of data that are characterized by early partitioning in the tree. The simplicity of the model and the interpretability of the RF method, the flexibility in using a large number of predictive variables and the limited sample size and their ability to pay attention to genetic heterogeneity have increased their application in genetic studies. In addition to prediction, it is involved in identifying very important variables [30].

SVMs are commonly used for issues where there are two categories. In this algorithm, the two-page classification is placed on the border of two data classes, and the problem is to find the maximum boundary between these two pages and, as a result, between the two categories of data. Accordingly, two pages become so far from each other that they collide with the data. A SVM is a classification that is considered among the core methods of machine learning. SVM has a high generalizability accuracy. The main idea in SVM is that assuming that the classes can be separated linearly yields super-pages that can separate classes. In problems where the data is not linearly separable, using nonlinear cores, we map the data to a space with more dimensions so that they can be separated linearly in this new space. Different cores can be used for SVM, such as RBF and LINEAR, etc. SVMs are one of the most well-known methods for classifying data by providing a statistical model. One of the problems we face in providing a nonlinear vector classification machine is the way of defining the core and its related parameters. Well-known class of core functions, such as Polynomial, Gaussian and sigmoid, have been introduced that require the adjustment of parameters for optimal performance. The *Sequential minimal optimization* is one of the well-known methods for teaching this classification machine at a desirable time.

Logistics regression is a standard statistical model for modeling binary responses [31]. In this method, the probability of the response variable (interval of burn to the start of treatment) is modeled as a linear function of the independent variables. Slope parameters in a logistic model can be interpreted as odds ratios. Linear structure and simple interpretation, appropriate software wide scope are the most important advantages of LR model.

All models are fitted with the variables introduced in Table 1. 70 % of the data were used as training data and the remaining 30 % were used as test data. Statistical analysis was performed using R software.

### Implementation and Performance Criteria

In this study, we classified the data into two categories randomly: training data and test data. 70 % of the data were used as training data and the remaining 30 % were used as test data. This process was repeated 100 times. Then, the mean of obtained sensitivity, specificity, overall accuracy, positive and negative predictive value, and positive and negative likelihood ratio from these 100 repetitions was used to compare the models. Classification models indicate the importance of a variable based on the percentage increase in the prediction error. A variable is selected as the most important if it creates the most error when it is removed. After scoring the importance of variables, they are ranked based on their importance.

## Results

In this study, 47.8 % of patients were under 14 years of age. 54.2 % of them were men, 52.7 % were married, and 78.9 % were unemployed. Also, half of the participants (50.6 %) had income < 200$. Further information is provided by the two groups of response variables in Table 1.

Table 2 show the most important factors associated with the distance between burns and the start of treatment, based on DT, RF and SVMs. Common variables between these models are: place of occurrence, time of the accident, location of transfer to hospital and income.

**Table 2:** Important factors based on data mining models (RF, SVM and DT)

Variables importance order	RF	SVM	DT
1	Place of occurrence	Time of accident	Location of transfer to hospital
2	Degree of burn	Age	Degree of burn
3	Province of residence	Location of transfer to hospital	Time of accident
4	Place of residence	Place of residence	Religion
5	Religion	Income	Place of occurrence
6	Income	Gender	Income
7	Location of transfer to hospital	Place of occurrence	Province of residence
8	Burnt limbs	Occupational status	Burnt limbs
9	Occupational status	History of burn	Education
10	Time of accident	Cause of burn	History of burn

### Model comparison

Table 3 represents the performance of different models. In the DT model, indicators such as total accuracy, sensitivity, positive likelihood ratio, negative predictive value and positive predictive value were higher than other models. However, the specificity of the SVM model and RF model was higher than other models, and the mean negative likelihood ratio of the SVM model are higher than other models.

**Table 3:** The performance of four models

Models	Total accuracy		Sensitivity		Specificity		Positive likelihood ratio		Negative likelihood ratio		Positive predictive value		Negative predictive value	
	Mean	Std. dev	Mean	Std. dev	Mean	Std. dev	Mean	Std. dev	Mean	Std. dev	Mean	Std. dev	Mean	Std. dev
**DT**	0.87	0.02	0.44	0.08	0.96	0.01	14.58	9.91	0.58	0.05	0.71	0.01	0.89	0.02
**RF**	0.82	0.03	0.05	0.03	0.99	0.01	6.09	4.87	0.96	0.03	0.50	0.16	0.83	0.03
**SVM**	0.83	0.03	0.02	0.004	0.99	0.0002	4.72	0.94	0.98	0.004	0.5	0	0.82	0.03
**LR**	0.83	0.02	0.19	0.09	0.96	0.02	7.08	7.49	0.84	0.09	0.48	0.25	0.85	0.02

## Discussion

In this study, data mining and LR techniques have been used to investigate the factors affecting the interval of burn to the start of treatment in burn patients in Imam Khomeini Hospital of Kermanshah city. Different models yielded different results, but in most indicators, the DT model performed better. In the DT model, indicators such as total accuracy, sensitivity, positive likelihood ratio, negative predictive value and positive predictive value were higher than other models. However, the specificity of the SVM model and RF model was higher than other models, and the mean negative likelihood ratio of the SVM model are higher than other models. According to the results of DT model, the most important factors affecting the interval of burn to start of treatment were location of transfer to hospital, degree of burn, time of accident, religion, place of occurrence, income, province of residence, burnt limbs, education, and history of burn. The time interval between burn and the start of treatment is one of the most important factors in the treatment process of burn patients, and no comprehensive study has been conducted so far [14, 15, 32]. Machine learning methods, especially the DT model, performed well in studies [33–38] in the field of burns and other areas of health science. According to the results of this study and the fact that no similar study has been done in this case, the variables of location of transfer to hospital, degree of burn, time of accident, religion, place of occurrence, income, province of residence, burnt limbs, education, history of burn were identified as important variables. The results of this study had two limitations. First limitation is related to cross-sectional design and observed associations did not show the causality. The second limitation of our study was that due to the lack of sufficient literature on the interval between burns and initiation of treatment in burn patients, it was not possible to compare the clinical results of this study with other studies.

## Conclusions

The results of current study indicated that DT model is better than data mining models in determining the effective factors in the interval between a burn and start of treatment in burn patients. Finding showed the most important factors affecting the interval of burn to start of treatment were location of transfer to hospital, degree of burn, time of accident, religion, place of occurrence, income, province of residence, burnt limbs, education, and history of burn.

## Data Availability

To access the data, he/she should coordinate with Imam Khomeini Hospital, which is under the supervision of Kermanshah University of Medical Sciences. Also, the reader can contact the first author and the corresponding author via the following emails: dr.ahmadi_jouybari@yahoo.com. kh.najafi420@yahoo.com.

## Abbreviations

CART: Classification and Regression Trees
DT: Decision Tree
SVM: Support Vector Machine
LR: Logistic Regression
RF: Random Forest
TBSA: The Total Body Surface Area
